# Recommendations of the Brazilian Society of Nephrology for sustainable nephrology in Brazil

**DOI:** 10.1590/2175-8239-JBN-2026-0059en

**Published:** 2026-07-24

**Authors:** Ana Flavia Moura, Cinthia Kruger Sobral Vieira, André Luiz Pimentel, Luis Claudio Santos Pinto, Fabiana Baggio Nerbass, Fátima Regina Veiga Tostes, Nandressa Dayana Mendes Riso, Rafael Barboza Trancoso, Talita Gavioli Salani, Ciro Bruno Silveira Costa, Patrícia Ferreira Abreu, José A Moura-Neto

**Affiliations:** 1 Sociedade Brasileira de Nefrologia, São Paulo, SP, Brazil.; 2 Escola Bahiana de Medicina e Saúde Pública, Salvador, BA, Brazil.; 3 Clinefro Serviço de Nefrologia e Diálise, Porto Alegre, RS, Brazil.; 4 Associação Brasileira dos Centros de Diálise e Transplante, Brasília, Brazil.; 5 Universidade Federal do Pará, Belém, PA, Brazil.; 6 Fundação Pró-Rim, Santa Catarina, SC, Brazil.; 7 Hospital São Vicente de Paulo, Rio de Janeiro, RJ, Brazil.; 8 Hospital Universitário do Oeste do Paraná, Paraná, PR, Brazil.; 9 Hospital Santa Rita AFECC, Vitória, ES, Brazil.; 10 Universidade Federal de São Paulo, São Paulo, SP, Brazil

**Keywords:** Nephrology, Dialysis, Sustainable Growth, Climate Change, Green Dialysis.

## Abstract

Climate change has increased the vulnerability of patients with non-dialysis chronic kidney disease (CKD) and those receiving kidney replacement therapy (KRT), who depend on healthcare systems that are highly sensitive to disruptions in electricity, potable water supply, and logistics. At the same time, KRT services are among those with the greatest environmental impact within the healthcare sector, characterized by high water and energy consumption and substantial generation of solid waste. In this context, the incorporation of sustainable practices has become an ethical, clinical, and strategic imperative. In 2023, the Brazilian Society of Nephrology (SBN) established the Committee on Sustainable Nephrology with the objective of developing national guidelines to reduce the environmental impact of kidney care services. After two years of work, the Committee developed ten recommendations structured across key domains, including education, environ­mental indicator monitoring, waste management, efficient use of resources, climate contingency planning, promotion of prevention and kidney transplantation, technological innovation, and sustainability certifications. These recommendations were discussed and approved at the 2nd Convention of the SBN Board of Directors and its Regional Chapters, achieving a 98.5% level of agreement. This document presents the consolidated recommendations, with the aim of supporting the implemen­tation of sustainable practices in kidney care services across the country and pro­moting an environmentally responsible, efficient nephrology practice aligned with the emerging challenges posed by climate change.

## Introduction

Climate change represents a growing threat to human health and to the functioning of healthcare systems on a global scale. The 2023 Synthesis Report of the Intergovernmental Panel on Climate Change^1^ demonstrated that global warming intensifies the occurrence of extreme events — such as heatwaves, prolonged droughts, floods, and wildfires — and increases the burden of infectious diseases, water- and foodborne illnesses, as well as morbidity and mortality related to heat stress. These phenomena have direct implications for kidney health. Recent reviews describe that exposure to heat and dehydration is associated with a higher risk of acute kidney injury, accelerated progression of chronic kidney disease (CKD), and increased incidence of occupational nephropathies in hot environments, as well as a possible increase in nephrolithiasis in high-temperature regions^2^,^3^,^4^.

In addition to the environmental effects on the occurrence and progression of CKD, nephrology faces another critical challenge: ensuring the continuity of kidney replacement therapies (KRT) in the context of environmental disasters. Patients undergoing hemodialysis (HD) and peritoneal dialysis (PD) are among the groups most vulnerable to disruptions in power supply, scarcity of potable water, transportation failures, flooding, and population displacement. Studies on natural disasters, including floods, hurricanes, and earthquakes, have demonstrated increased hospitalizations, mortality, and the need for specific preparedness strategies to ensure the delivery of dialysis treatments during crises, highlighting the need for more resilient and integrated response systems^5^,^6^,^7^.

Paradoxically, KRT services are among those with the greatest environmental impact within the healthcare sector. Conventional HD requires large volumes of water and energy and generates substantial amounts of solid waste, including dialyzers, tubing, cartridges, plastic packaging, and concentrated solutions. Life-cycle analyses and estimates from HD centers suggest that a single patient may consume approximately 60,000 to 80,000 liters of water per year, considering three weekly sessions, and generate more than 300 kg of solid waste annually, in addition to significant energy consumption associated with dialysis machines and auxiliary systems^8^,^9^,^10^. In the Brazilian context, published estimates indicate that dialysis units collectively consume billions of liters of water and generate tens of thousands of tons of waste each year, reinforcing the need to integrate envi­ronmental considerations into healthcare planning^10^.

In this scenario, large-scale simulation models have contributed to quantifying the clinical, economic, and environmental impact of CKD. The IMPACT CKD study, a patient-level micro­simulation model, projected the burden of CKD across eight countries — including Brazil — over a ten-year period, incorporating clinical outcomes, healthcare resource utilization, costs, and environmental impacts^11^. The analysis demonstrated that, if the current pattern of late diagnosis and suboptimal management persists, there will be a substantial increase in CKD prevalence and in the number of individuals in advanced stages by 2032, leading to a corresponding rise in demand for KRT, as well as increased greenhouse gas emissions, freshwater use, and fossil fuel consumption associated with kidney care^11^,^12^. Complementary studies indicate that strategies focused on prevention, early diagnosis, and greater adherence to nephroprotective therapies can substantially reduce the need for dialysis, cardiovascular events, and the environmental impact related to CKD management^12^,^13^.

The discussion on the carbon footprint of the healthcare sector has also gained prominence. A global analysis estimated that healthcare systems account for more than 4% of worldwide greenhouse gas emissions, with significant contributions from medications, medical devices, and supply chains^14^. In nephrology, dedicated reviews highlight dialysis as one of the components with the highest envi­ronmental impact, due to the combined effects of water and energy consumption, waste generation, and emissions associated with the transport of patients and supplies^8^,^9^,^10^,^15^.

Progress toward more sustainable nephrology requires technological modernization, more efficient care practices, and institutional policies that promote waste reduction, responsible resource use, and appropriate waste management. It also involves increased investment in primary and secondary prevention of CKD, expansion of access to kidney transplantation, and the adoption of care models that reduce dependence on high-impact therapies^10^,^11^,^15^.

**Figure 1:**
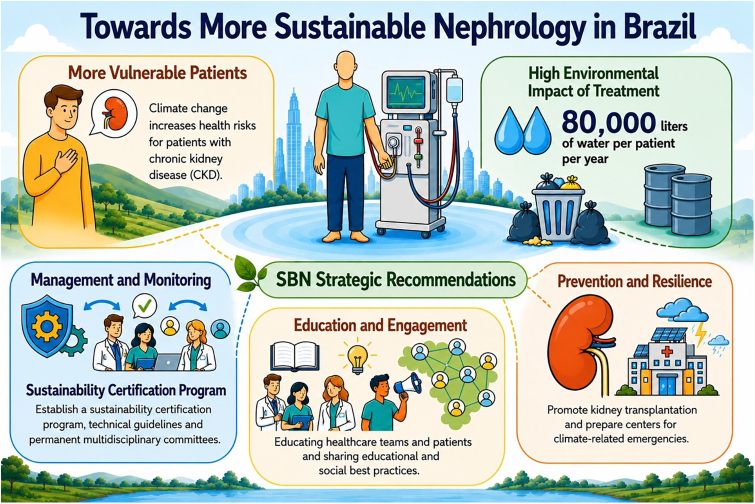
Environmental challenges and pathways toward sustainable nephrology in Brazil.

Brazilian nephrology must incorporate environ­mentally responsible practices as part of a new model of care, in which sustainability is recognized as an ethical, clinical, and strategic pillar of kidney care^10^ (Figure 1). Recognizing this urgency, the Brazilian Society of Nephrology (*Sociedade Brasileira de Nefrologia*, SBN) established, in 2023, the Committee on Sustainable Nephrology with the purpose of guiding, coordinating, and promoting sustainable practices in Brazilian nephrology. During the 2023–2024 biennium, the Committee developed national recommendations, which were presented at the 2nd Convention of the SBN Board of Directors and its Regional Chapters, held in February 2025, consolidating an institutional commitment to building a more sustainable nephrology in Brazil.

## Methodology for the Development of the Recommendations

The recommendations presented in this document were developed by the SBN Committee on Sustainable Nephrology through a structured, collaborative process grounded in scientific evidence. Initially, a narrative review of the national and international literature on climate change, the environmental impact of nephrology, and sustainability strategies in healthcare was conducted, with emphasis on the studies and references previously cited in the “Introduction” section of this statement.

In parallel, the Committee promoted internal discussions aimed at integrating scientific evidence, practical experience, and the Brazilian healthcare context. Preliminary proposals were organized into strategic domains, which were subsequently consolidated into the ten recommendations presented herein.

The final set of recommendations was submitted for review and deliberation at the 2nd Convention of the SBN Board of Directors and its Regional Chapters, held in São Paulo on February 14–15, 2025. This meeting brought together members of the SBN national board, representatives from the leadership of active SBN Regional Chapters, and directors of SBN Departments. In total, 39 participants were involved. Of the ten proposed recommendations, seven were approved unanimously (100%), and three received broad acceptance (93.8%–97.1%). A total of 34 qualitative comments were received and subsequently reviewed by the Committee.

It is recognized that the implementation of these recommendations should occur progressively and be adapted to different regional contexts. Specific indicators for monitoring and evaluation of each domain will be discussed, defined, and validated in a subsequent phase by a panel of experts, ensuring technical rigor, operational feasibility, and alignment with international best practices.

## Recommendations

The following section presents the SBN Recommendations for Sustainable Nephrology in Brazil. This section constitutes the strategic core of the document and aims to provide practical guidance for the progressive incorporation of environmental sustainability into nephrology practice in Brazil, while respecting the country’s regional, structural, and healthcare differences. Table 1 summarizes the ten recommendations, outlining the objectives and strategic actions for each, as well as the stakeholders responsible for monitoring their implementation.

**Table 1:** SBN recommendations for sustainable nephrology in brazil

Recommendation	Objective	Strategic actions	Responsible parties
1. Sustainable Nephrology Seal	Recognize and promote sustainable practices in nephrology centers.	Define measurable criteria; monitor water, energy, and waste consumption; establish local committees.	Sustainable Nephrology Committee | SBN
2. Environmental Education and Awareness	Train professionals and patients in sustainable kidney care practices.	Develop training programs and workshops; conduct ongoing campaigns; encourage active participation.	Sustainable Nephrology Committee | SBN
3. Sharing of Best Practices	Promote the exchange of sustainable experiences among the centers.	Organize conferences; publish success cases and reports.	SBN | Sustainable Nephrology Committee
4. Regional Ambassadors	Promote sustainability in a decentralized manner across the country.	Appoint regional ambassadors; establish local committees; recognize regional initiatives.	Sustainable Nephrology Committee | SBN Regional Chapters
5. Multidisciplinary Committees	Strengthen team engagement in sustainable practices.	Support the creation of internal committees; train multidisciplinary teams.	Sustainable Nephrology Committee | Dialysis Centers
6. Community Engagement	Expand the involvement of patients, families, and communities.	Create support networks; encourage community participation; develop educational actions.	Local Committees | Community Leaders | SBN
7. Technical Document for Dialysis Centers	Provide practical sustainability guidelines.	Develop a technical document; promote clean energy, water conservation, and waste management.	Sustainable Nephrology Committee
8. Sustainability in SBN Initiatives	Integrate sustainable practices into institutional activities.	Adopt environmental criteria in events; reduce printed materials; pursue carbon neutrality.	SBN National Board
9. Climate Contingency Document	Ensure the continuity of kidney care during disasters.	Develop contingency protocols; train teams and communities.	Sustainable Nephrology Committee | SBN | Ministry of Health
10. CKD Prevention and Kidney Transplantation	Reduce dependence on dialysis through prevention and transplantation.	Expand screening; strengthen primary care; promote organ donation and transplantation.	SBN | SBN Regional Chapters

Abbeviations – SBN, Brazilian Society of Nephrology; CKD, Chronic Kidney Disease.

### Implementation of the Sustainable Nephrology Seal

The establishment of an SBN Sustainable Nephrology Seal aims to recognize, encourage, and provide institutional visibility to nephrology units that adopt environmentally responsible practices. International experience demonstrates that certification and recognition systems are effective tools to drive organizational change, standardize best practices, and promote continuous improvement in healthcare services with high environmental impact, such as dialysis^8^.

In the context of nephrology, where water and energy consumption and waste generation are substantial, an official SBN seal may serve as an educational, regulatory, and symbolic instrument, encouraging centers to monitor their processes and reduce waste. Its practical implementation involves the definition of progressive, auditable criteria adapted to the Brazilian context, allowing units of different sizes to advance across levels of sustainability. The environmental indicators associated with the seal will be defined at a later stage by a specialized technical committee, ensuring methodological rigor, comparability, and operational feasibility.

### Environmental Education and Awareness

Education is a central pillar in the transition toward sustainable nephrology. Evidence shows that lasting changes in healthcare practices depend on the environmental awareness of healthcare professionals, the engagement of multidisciplinary teams, and the active participation of patients^8^.

This recommendation proposes the systematic incorporation of environmental sustainability content into nephrology, including the ecological impact of dialysis, the rational use of resources, and the relationship between climate change and kidney disease. In practice, this translates into the provision of in-person and virtual training, workshops, sustainable care protocols, and ongoing educational campaigns within healthcare units. Patients and their families should also be included in this process, fostering a shared culture of environmental responsibility aligned with patient-centered care.

### Sharing of Best Practices

The heterogeneity of nephrology services in Brazil is substantial, but it also represents an opportunity for collective learning. Several centers have already implemented isolated sustainability initiatives, often involving simple, low-cost solutions with meaningful impact^10^,^16^.

The structured sharing of these experiences can accelerate the dissemination of best practices, avoid duplication of efforts, and stimulate innovation. This recommendation includes the organization of periodic meetings — either in-person or virtual — dedicated to sustainability in nephrology, as well as the publication of experience reports, outcomes from the Sustainable Nephrology Seal, and technical reports. Such an exchange strengthens the development of a national community of practice in sustainable nephrology.

### Appointment of Regional Ambassadors

Given Brazil’s continental dimensions and regional inequalities, sustainability in nephrology requires a decentralized strategy. The appointment of regional ambassadors for sustainable nephrology aims to adapt national recommendations to local realities and to strengthen regional engagement.

These ambassadors will act as focal points, facilitating coordination among nephrology centers, the SBN Regional Chapters, and the Sustainability Committee. Their role includes providing technical support, encouraging the establishment of local committees, and recognizing successful regional initiatives while respecting climatic, social, and structural specificities.

### Establishment of Multidisciplinary Committees

Sustainability in healthcare requires a multidisci­plinary approach. In the context of nephrology, such strategies depend on the involvement of physicians, nurses, nursing technicians, psychologists, nutritionists, social workers, other healthcare professionals, clinical engineers, administrators, cle­aning staff, suppliers, patients, and their families^1^ ^7^,^18^.

This recommendation proposes that each nephrology center establish internal sustainability committees responsible for discussing, implementing, and monitoring environmental actions in daily practice. These committees strengthen a sense of shared responsibility, facilitate the identification of opportunities for improvement, and promote culturally sustainable changes that extend beyond isolated initiatives.

### Community Engagement

Patients receiving kidney replacement therapy and their families are among the groups most vulnerable to the effects of climate change and extreme environmental events^5^,^6^,^7^. At the same time, they are key stakeholders in the adoption and success of sustainable strategies.

This recommendation emphasizes the engagement of communities served by nephrology services, promoting environmental education, participation in sustainability initiatives, and the strengthening of support and resilience networks. Community involvement extends the impact of these actions beyond dialysis units, reinforcing the social role of nephrology and its connection with the local context. This approach is aligned with contemporary stakeholder-oriented models of care, in which patients, families, and communities are recognized as legitimate stakeholders and co-responsible for the sustainability of dialysis services^19^.

### Development of a Practical Guidance Document for Dialysis Centers

Although the literature on sustainable nephrology is expanding, many services still lack practical guidance adapted to local realities. Therefore, the development of a specific technical document with recommendations applicable to Brazilian dialysis centers is proposed.

This document should address key areas such as efficient water use, safe reuse practices, energy efficiency, waste management, sustainable procurement, and the incorporation of lower-impact technologies. Its purpose is to serve as an operational guide, supporting managers and healthcare teams in making evidence-based decisions.

### Implementation of Sustainable Practices in the SBN Institutional Initiatives

As a national representative organization, the SBN should lead by example. Incorporating sustainability criteria into its headquarters, congresses, scientific events, meetings, and publications reinforces institutional coherence and broadens the reach of environmental messages.

Practical implementation includes reducing the use of plastic and printed materials, prioritizing digital resources, selecting suppliers with responsible practices, and, whenever possible, adopting strategies for emissions mitigation and offsetting. These actions align the SBN’s institutional activities with the recommendations it promotes for healthcare services.

### Climate Contingency Document

Extreme events associated with climate change have a direct impact on the continuity of kidney care. Studies have demonstrated increased morbidity and mortality among dialysis patients during natural disasters, highlighting the need for specific planning^5^,^6^.

This recommendation proposes the development of a National Climate Contingency Document in Nephrology, including protocols for different crisis scenarios. The document should provide guidance to healthcare services, professionals, and patients regarding preparedness, response, and recovery and should be integrated with local health systems.

### Promotion of CKD Prevention and Kidney Transplantation

No sustainability strategy in nephrology is complete without adequate investment in CKD prevention and the expansion of access to kidney transplantation. Evidence demonstrates that prevention, early diag­nosis, and appropriate management of CKD reduce progression to advanced stages, decrease the need for dialysis, and consequently reduce the environmental impact of kidney care^11^.

Kidney transplantation, in addition to offering improved quality of life and survival, has a lower environmental footprint compared with dialysis. Therefore, this recommendation reinforces the role of the SBN in promoting prevention programs, screening, early diagnosis, and CKD management, as well as strengthening primary care capacity, educational campaigns, and policies supporting organ donation and transplantation.

## Final Considerations

The ten recommendations described herein constitute an integrated strategic framework for the development of environmentally sustainable nephrology in Brazil. Their implementation should be progressive and phased over a four-year period (2027–2030), grounded in evidence and adapted to the country’s diverse regional contexts. Specific indicators for each domain will be discussed, defined, and validated by a panel of experts, ensuring transparency, technical rigor, and applicability.

By adopting these recommendations, Brazilian nephrology advances in the alignment of clinical excellence, environmental responsibility, and social commitment, positioning itself proactively to address the challenges posed by climate change and the growing burden of chronic kidney disease.

## Data Availability

The datasets generated and/or analyzed during the current study are available from the corresponding author upon reasonable request.

## References

[1] Intergovernmental Panel on Climate Change (2023). Climate change 2023: synthesis report [Internet].

[2] Johnson RJ, Sánchez-Lozada LG, Newman LS, Lanaspa MA, Diaz HF, Lemery J (2019). Climate change and the kidney. Ann Nutr Metab.

[3] Qu Y, Zhang W, Boutelle AM, Ryan I, Deng X, Liu X (2023). Associations between ambient extreme heat exposure and emergency department visits related to kidney disease. Am J Kidney Dis.

[4] Hess HW, Stooks JJ, Baker TB, Chapman CL, Johnson BD, Pryor RR (2022). Kidney injury risk during prolonged exposure to current and projected wet bulb temperatures occurring during extreme heat events in healthy young men. J Appl Physiol.

[5] Sapkota A, Kotanko P (2023). Climate change-fuelled natural disasters and chronic kidney disease: a call for action. Nat Rev Nephrol.

[6] Silva DR, Luz LGD, Moura-Neto JA (2025). Lessons from the catastrophic floods in southern Brazil: geographic and environmental factors, vulnerabilities, and the resilience of nephrology services during natural disasters. Curr Opin Nephrol Hypertens.

[7] Yoo KD, Kim HJ, Kim Y, Park JY, Shin SJ, Han SH (2019). Disaster preparedness for earthquakes in hemodialysis units in Gyeongju and Pohang, South Korea. Kidney Res Clin Pract.

[8] Barraclough KA, Agar JWM (2020). Green nephrology. Nat Rev Nephrol.

[9] Saleem S, Stigant C, Rajan T, Hewage K, Sadiq R, MacNeill AJ (2025). Environmental impacts of kidney replacement therapies: a comparative lifecycle assessment. Am J Kidney Dis.

[10] Moura-Neto JA, Barraclough K, Agar JW (2019). Green nephrology: a call to action. J Bras Nefrol.

[11] Brown S, Garcia Sanchez JJ, Guiang H, Priest S, Wheeler DC, Moura AF (2024). IMPACT CKD: holistic disease model projecting 10-year population burdens. Kidney Int Rep.

[12] Rao N, Brotons-Munto F, Moura AF, Kocks JWH, Zhao MH, Chadban S (2025). Holistic Impact of CKD: a clinical, economic, and environmental analysis by IMPACT CKD. Kidney Int Rep.

[13] Tangri N, Priest S, Zara A, Long BR, Chen J, Rao N (2025). Impact of improved diagnosis and treatment on holistic CKD burden. Kidney Int Rep.

[14] Lenzen M, Malik A, Li M, Fry J, Weisz H, Pichler PP (2020). The environmental footprint of health care: a global assessment. Lancet Planet Health.

[15] Otero González A (2024). Chronic kidney disease, dialysis and climate change. Nefrologia (Engl Ed).

[16] Nerbass FB, Schulz R, Vargas MA, Machado MT, Vieira MA (2025). Green nephrology in practice: actions that promote environmental, social, and economic impact. J Bras Nefrol.

[17] Stigant CE, Rajan T, Barraclough KA, Miller FA (2023). The necessity of environmentally sustainable kidney care. Can J Kidney Health Dis.

[18] Mortimer F, Isherwood J, Wilkinson A, Vaux E (2018). Sustainability in quality improvement: redefining value. Future Healthc J.

[19] Moura-Neto JA (2025). Non sibi sed omnibus: chronic dialysis care through a stakeholder lens. Blood Purif.

