# Supplementary material for: Recommendations of the Brazilian Society of Nephrology for sustainable nephrology in Brazil
Source: J Bras Nefrol. 2026 Jul 24;48(3):e20260059. doi: 10.1590/2175-8239-JBN-2026-0059en (PMC13427308; doi:10.1590/2175-8239-JBN-2026-0059en)
Supplement: Translation

# Translation: Recomendações da Sociedade Brasileira de Nefrologia por uma nefrologia sustentável no Brasil

**DOI:** 10.1590/2175-8239-JBN-2026-0059pt

**Authors:** Ana Flavia Moura, Cinthia Kruger Sobral Vieira, André Luiz Pimentel, Luis Claudio Santos Pinto, Fabiana Baggio Nerbass, Fátima Regina Veiga Tostes, Nandressa Dayana Mendes Riso, Rafael Barboza Trancoso, Talita Gavioli Salani, Ciro Bruno Silveira Costa, Patrícia Ferreira Abreu, José A Moura-Neto

**Affiliations:** 1 Sociedade Brasileira de Nefrologia, São Paulo, SP, Brazil.; 2 Escola Bahiana de Medicina e Saúde Pública, Salvador, BA, Brazil.; 3 Clinefro Serviço de Nefrologia e Diálise, Porto Alegre, RS, Brazil.; 4 Associação Brasileira dos Centros de Diálise e Transplante, Brasília, Brazil.; 5 Universidade Federal do Pará, Belém, PA, Brazil.; 6 Fundação Pró-Rim, Santa Catarina, SC, Brazil.; 7 Hospital São Vicente de Paulo, Rio de Janeiro, RJ, Brazil.; 8 Hospital Universitário do Oeste do Paraná, Paraná, PR, Brazil.; 9 Hospital Santa Rita AFECC, Vitória, ES, Brazil.; 10 Universidade Federal de São Paulo, São Paulo, SP, Brazil

**Keywords:** Nefrologia, Diálise, Crescimento Sustentável, Mudança Climática, Diálise Verde.

## Abstract

As mudanças climáticas têm ampliado a vulnerabilidade de pacientes com doença renal crônica (DRC) não dialítica e daqueles em terapia renal substitutiva (TRS), os quais dependem de sistemas de saúde sensíveis a interrupções no fornecimento de energia, água potável e logística. Paralelamente, os serviços de TRS figuram entre os de maior impacto ambiental dentro do setor da saúde, caracterizados por elevado consumo de água e energia e pela geração expressiva de resíduos sólidos. Diante desse cenário, a incorporação de práticas sustentáveis tornou-se um imperativo ético, assistencial e estratégico. Em 2023, a Sociedade Brasileira de Nefrologia (SBN) instituiu o Comitê de Sustentabilidade em Nefrologia, com o objetivo de delinear diretrizes nacionais para reduzir o impacto ambiental dos serviços renais. Após dois anos de trabalho, o Comitê desenvolveu dez recomendações estruturadas nos seguintes eixos: educação, monitoramento de indicadores ambientais, gestão de resíduos, uso eficiente de recursos, planos de contingência climática, incentivo à prevenção e ao transplante renal, inovação tecnológica e certificações de sustentabilidade. As recomendações foram discutidas e aprovadas na 2ª Convenção da Diretoria Plena da SBN e suas Regionais, alcançando 98,5% de concordância. Este documento apresenta as recomendações consolidadas, com o propósito de apoiar a implementação de práticas sustentáveis nos serviços renais em todo o país e promover uma nefrologia ambientalmente responsável, eficiente e alinhada aos desafios emer­gentes impostos pelas mudanças climáticas.

## Introdução

As mudanças climáticas representam uma ameaça crescente à saúde humana e ao funcionamento dos sistemas de saúde em escala global. O Relatório de Síntese do Painel Intergovernamental sobre Mudanças Climáticas, de 2023^1^, demonstrou que o aquecimento global intensifica a ocorrência de eventos extremos — como ondas de calor, secas prolongadas, enchentes e queimadas — e aumenta a carga de doenças infecciosas, enfermidades transmitidas por água e alimentos, bem como a morbimortalidade relacionada ao estresse térmico. Esses fenômenos têm repercussões diretas sobre a saúde renal. Revisões recentes descrevem que a exposição ao calor e à desidratação está associada a um maior risco de lesão renal aguda, aceleração da progressão da doença renal crônica (DRC) e maior incidência de nefropatias relacionadas ao trabalho em ambientes quentes, além de possível aumento da nefrolitíase em regiões de temperaturas elevadas^2^,^3^,^4^.

Além dos efeitos ambientais sobre a ocorrência e progressão da DRC, a nefrologia enfrenta outro desafio crítico: garantir a continuidade das terapias renais substitutivas (TRS) em cenários de desastre ambiental. Pacientes em hemodiálise (HD) e diálise peritoneal (DP) estão entre os grupos mais vulneráveis a interrupções de energia, escassez de água potável, falhas de transporte, enchentes e deslocamentos populacionais. Estudos sobre desastres naturais, incluindo enchentes, furacões e terremotos, mostram aumento de hospitalizações, mortalidade e necessidade de estratégias específicas de preparação para assegurar a realização das sessões de diálise em situações de crise, evidenciando a necessidade de sistemas de resposta mais resilientes e integrados^5^,^6^,^7^.

Paradoxalmente, os serviços de TRS possuem um dos principais impactos ambientais no setor da saúde. A HD convencional utiliza grandes volumes de água e energia e gera quantidades substanciais de resíduos sólidos, provenientes de dialisadores, linhas, cartuchos, embalagens plásticas e soluções concentradas. Análises de ciclo de vida e estimativas realizadas em centros de HD sugerem que um paciente pode consumir em torno de 60.000 a 80.000 litros de água por ano, considerando três sessões semanais, e gerar mais de 300 kg de resíduos sólidos anuais, além de elevado consumo energético associado ao funcionamento das máquinas e dos sistemas auxiliares^8^,^9^,^10^. No contexto brasileiro, estimativas publicadas indicam que o conjunto das unidades de diálise pode utilizar bilhões de litros de água e produzir dezenas de milhares de toneladas de resíduos por ano, reforçando a necessidade de integrar a discussão ambiental ao planejamento assistencial^10^.

Nesse cenário, modelos de simulação em larga escala têm contribuído para quantificar o impacto clínico, econômico e ambiental da DRC. O estudo IMPACT CKD, um modelo de microssimulação em nível de paciente, projetou a carga de DRC em oito países — incluindo o Brasil — ao longo de dez anos, incorporando desfechos clínicos, uso de recursos em saúde, custos e impactos ambientais^11^. A análise demonstrou que, mantido o padrão atual de diagnóstico tardio e manejo subótimo, haverá aumento expressivo na prevalência de DRC e no número de indivíduos em estágios avançados até 2032, com consequente crescimento da demanda por TRS e das emissões de gases de efeito estufa, do uso de água doce e do consumo de combustíveis fósseis associados ao cuidado renal^11^,^12^. Estudos complementares indicam que estratégias de prevenção, diagnóstico precoce e maior adesão às terapias nefroprotetoras podem reduzir substancialmente a necessidade de diálise, eventos cardiovasculares e o impacto ambiental relacionado ao manejo da DRC^12^,^13^.

A discussão sobre a pegada de carbono do setor da saúde também ganhou relevância. Uma análise global estimou que os sistemas de saúde respondem por mais de 4% das emissões mundiais de gases de efeito estufa, com grande contribuição de medicamentos, dispositivos médicos e cadeias de suprimento^14^. Em nefrologia, revisões dedicadas destacam a diálise como um dos componentes de maior impacto ambiental, pela combinação de consumo de água, energia, produção de resíduos e emissões associadas ao transporte de pacientes e insumos^8^,^9^,^10^,^15^.

O avanço rumo a uma nefrologia mais sustentável requer modernização tecnológica, práticas assis­tenciais mais eficientes e políticas institucionais que promovam a redução de desperdícios, o uso inteligente de recursos e a gestão adequada de resíduos. Envolve também maior investimento em prevenção primária e secundária da DRC, ampliação do acesso ao transplante renal e adoção de modelos de cuidado que reduzam a dependência de terapias de alto impacto ambiental^10^,^11^,^15^.

A nefrologia brasileira precisa incorporar práticas ambientais responsáveis como parte de um novo modelo de cuidado, no qual a sustentabilidade seja reconhecida como um pilar ético, clínico e estratégico do tratamento renal^10^ (Figura 1). Reconhecendo essa urgência, a Sociedade Brasileira de Nefrologia (SBN) instituiu, em 2023, o Comitê de Sustentabilidade em Nefrologia com o propósito de orientar, articular e promover práticas sustentáveis na nefrologia brasileira. No biênio 2023–2024, o Comitê desenvolveu recomendações nacionais, que foram apresentadas na 2ª Convenção da Diretoria Plena da SBN e suas Regionais, realizada em fevereiro de 2025, consolidando um compromisso institucional com a construção de uma nefrologia mais sustentável no Brasil.

**Figura 1:** Desafios ambientais e caminhos para uma nefrologia sustentável no Brasil.

## Metodologia de Elaboração das Recomendações

As recomendações apresentadas neste documento foram desenvolvidas pelo Comitê de Sustentabilidade em Nefrologia da SBN ao longo de um processo estruturado, colaborativo e fundamentado em evidências científicas. Inicialmente, foi realizada uma revisão narrativa da literatura nacional e internacional sobre mudanças climáticas, impacto ambiental da nefrologia e estratégias de sustentabilidade em saúde, com ênfase nos estudos e referências já citados na seção “Introdução” deste posicionamento.

Em paralelo, o Comitê promoveu discussões internas buscando integrar evidências científicas, experiências práticas e a realidade assistencial brasileira. As propostas preliminares foram organizadas em eixos estratégicos, posteriormente consolidados nas dez recomendações aqui apresentadas.

O conjunto final de recomendações foi submetido à apreciação e deliberação da 2ª Convenção da Diretoria Plena da SBN e suas Regionais, realizada em São Paulo, em 14 e 15 de fevereiro de 2025. Esse encontro reuniu membros da diretoria nacional da SBN, representantes da diretoria das Regionais ativas da instituição e diretores dos Departamentos da SBN. Ao todo, foram registrados 39 participantes. Das dez recomendações propostas, sete foram aprovadas por unanimidade (100%) e três obtiveram ampla aceitação (93,8% a 97,1%). Foram recebidos 34 comentários qualitativos, avaliados posteriormente pelo Comitê.

Reconhece-se que a implementação das recomen­dações deverá ocorrer de forma progressiva e adaptada aos diferentes contextos regionais. Os indicadores específicos de monitoramento e avaliação de cada eixo serão discutidos, definidos e validados em etapa subsequente por um comitê de especialistas, garantindo rigor técnico, viabilidade operacional e alinhamento com as melhores práticas internacionais.

## Recomendações

A seguir, são apresentadas as Recomendações da SBN por uma nefrologia sustentável no Brasil. Esta seção constitui o núcleo estratégico do documento e tem como finalidade orientar, de forma prática, a incorporação progressiva da sustentabilidade ambiental à prática nefrológica brasileira, respeitando as diferenças regionais, estruturais e assistenciais do país. A Tabela 1 resume as dez recomendações, descrevendo o objetivo e as ações estratégicas de cada uma delas, bem como os responsáveis pela monitorização dessas estratégias.

**Tabela 1:** Recomendações da SBN para uma nefrologia sustentável no Brasil

Recomendação	Objetivo	Ações estratégicas	Responsáveis
1. Selo de Nefrologia Sustentável	Reconhecer e incentivar práticas sustentáveis nos centros de nefrologia.	Definir critérios mensuráveis; monitorar consumo de água, energia e resíduos; instituir comissão local.	Comitê de Nefrologia Sustentável | SBN
2. Educação Ambiental e Conscientização	Capacitar profissionais e pacientes para práticas sustentáveis no cuidado renal.	Desenvolver treinamentos e workshops; realizar campanhas contínuas; estimular participação ativa.	Comitê de Nefrologia Sustentável | SBN
3. Compartilhamento de Boas Práticas	Estimular o intercâmbio de experiências sustentáveis entre os centros.	Organizar conferências; publicar casos de sucesso e relatórios.	Comitê de Nefrologia Sustentável | SBN
4. Embaixadores Regionais	Promover a sustentabilidade de forma capilarizada no país.	Designar embaixadores regionais; criar comissões locais; reconhecer iniciativas regionais.	Comitê de Nefrologia Sustentável | Regionais da SBN
5. Comissões Multidisciplinares	Fortalecer o engajamento das equipes em práticas sustentáveis.	Apoiar a criação de comissões internas; capacitar equipes.	Comitê de Nefrologia Sustentável | Clínicas de Diálise
6. Engajamento das Comunidades	Ampliar o envolvimento de pacientes, familiares e comunidade.	Criar redes de apoio; estimular a participação comunitária; promover ações educativas.	Comissões Locais | Líderes Comunitários | SBN
7. Documento Técnico para Centros de Diálise	Fornecer diretrizes práticas de sustentabilidade.	Elaborar documento técnico; incentivar o uso de energia limpa, a redução do consumo de água e a gestão de resíduos.	Comitê de Nefrologia Sustentável
8. Sustentabilidade nas Iniciativas da SBN	Integrar práticas sustentáveis às atividades institucionais.	Adotar critérios ambientais em eventos; reduzir impressões; buscar neutralidade de carbono.	Diretoria da SBN
9. Documento de Contingência Climática	Assegurar a continuidade do cuidado renal em desastres.	Elaborar protocolos de contingência; capacitar equipes e comunidades.	Comitê de Nefrologia Sustentável | SBN | Ministério da Saúde
10. Prevenção da DRC e Transplante Renal	Reduzir a dependência da diálise por meio da prevenção e do transplante.	Ampliar rastreio; capacitar atenção básica; promover doação e transplante.	SBN | Regionais da SBN

Abreviaturas – SBN, Sociedade Brasileira de Nefrologia; DRC, Doença Renal Crônica.

### Implementação do Selo de Nefrologia Sustentável

A criação de um Selo de Nefrologia Sustentável da SBN visa reconhecer, estimular e dar visibilidade institucional às unidades de nefrologia que adotem práticas ambientalmente responsáveis. Experiências internacionais demonstram que sistemas de certificação e reconhecimento são ferramentas eficazes para induzir mudanças organizacionais, padronizar boas práticas e promover melhoria contínua em serviços de saúde de alto impacto ambiental, como a diálise^8^.

No contexto da nefrologia, onde o consumo de água, energia e a geração de resíduos são elevados, um selo oficial da SBN pode funcionar como instrumento educativo, regulatório e simbólico, incentivando centros a monitorar seus processos e a reduzir desperdícios. A aplicação prática envolve a definição de critérios progressivos, auditáveis e adaptados à realidade brasileira, permitindo que unidades de diferentes portes avancem em níveis de sustentabilidade. Os indicadores ambientais associados ao selo serão definidos posteriormente por um comitê técnico especializado, garantindo rigor metodológico, comparabilidade e viabilidade operacional.

### Educação Ambiental e Conscientização

A educação é um dos pilares centrais para a transição rumo a uma nefrologia sustentável. Evidências demonstram que mudanças duradouras em práticas assistenciais dependem da consciência ambiental dos profissionais de saúde, do engajamento das equipes multiprofissionais e da participação ativa dos pacientes^8^.

Esta recomendação propõe a incorporação sistemática de conteúdos sobre sustentabilidade ambiental na nefrologia, incluindo o impacto ecológico da diálise, o uso racional de recursos e a relação entre mudanças climáticas e doença renal. Na prática, isso se traduz na oferta de treinamentos presenciais e virtuais, workshops, protocolos assistenciais sustentáveis e campanhas educativas contínuas nas unidades. Pacientes e familiares também devem ser incluídos nesse processo, fortalecendo uma cultura compartilhada de responsabilidade ambiental, alinhada ao cuidado centrado no paciente.

### Compartilhamento de Boas Práticas

A heterogeneidade dos serviços de nefrologia no Brasil é ampla, mas também representa uma oportunidade de aprendizado coletivo. Diversos centros já adotam iniciativas sustentáveis isoladas, muitas vezes com soluções simples e de baixo custo, porém com impacto relevante^10^,^16^.

O compartilhamento estruturado dessas experiências permite acelerar a disseminação de boas práticas, evitar retrabalho e estimular a inovação. A recomendação inclui a organização de encontros periódicos — presenciais ou virtuais — dedicados à sustentabilidade em nefrologia, bem como a publicação de relatos de experiências, resultados do selo sustentável e relatórios técnicos. Essa troca fortalece a construção de uma comunidade de prática nacional em nefrologia sustentável.

### Nomeação de Embaixadores Regionais

Considerando as dimensões continentais do Brasil e suas desigualdades regionais, a sustentabilidade em nefrologia precisa de uma estratégia capilarizada. A nomeação de embaixadores regionais da nefrologia sustentável tem como objetivo adaptar as recomendações nacionais às realidades locais e fortalecer o engajamento regional.

Esses embaixadores atuarão como pontos focais, articulando centros de nefrologia, Regionais da SBN e o Comitê de Sustentabilidade. Sua atuação inclui apoio técnico, estímulo à criação de comissões locais e valorização de iniciativas regionais bem-sucedidas, respeitando especificidades climáticas, sociais e estruturais.

### Criação de Comissões Multidisciplinares

A sustentabilidade em saúde precisa ser multi­disciplinar. No contexto da nefrologia, estratégias como essa precisam do envolvimento de médicos, enfermeiros, técnicos de enfermagem, psicólogos, nutricionistas, assistentes sociais, outros profissionais de saúde, engenheiros clínicos, gestores, equipes de limpeza, fornecedores, pacientes e familiares^17^,^18^.

Essa recomendação propõe que cada centro de nefrologia constitua comissões internas de susten­tabilidade, responsáveis por discutir, implementar e monitorar ações ambientais no cotidiano do serviço. Essas comissões fortalecem o senso de corresponsabilidade, facilitam a identificação de oportunidades de melhoria e promovem mudanças culturalmente sustentáveis, indo além de ações pontuais.

### Engajamento das Comunidades

Pacientes em terapia renal substitutiva e suas famílias estão entre os grupos mais vulneráveis aos efeitos das mudanças climáticas e de eventos ambientais extremos^5^,^6^,^7^. Ao mesmo tempo, são atores fundamentais para a adesão e o sucesso das estratégias sustentáveis.

Esta recomendação enfatiza o engajamento das comunidades atendidas pelos serviços de nefrologia, promovendo educação ambiental, participação em ações sustentáveis e o fortalecimento de redes de apoio e resiliência. O envolvimento comunitário amplia o impacto das ações para além das unidades de diálise, reforçando o papel social da nefrologia e sua conexão com o território. Essa abordagem está alinhada ao modelo de cuidado contemporâneo orientado por *stakeholders*, no qual pacientes, famílias e comunidade são reconhecidos como partes interessadas legítimas e corresponsáveis pela sustentabilidade dos serviços de diálise^19^.

### Documento de Recomendações Práticas Para Centros de Diálise

Embora a literatura sobre nefrologia sustentável esteja em expansão, muitos serviços ainda carecem de diretrizes práticas adaptadas à realidade local. Assim, recomenda-se a elaboração de um documento técnico específico, com orientações aplicáveis aos centros de diálise brasileiros.

Esse documento deverá abordar temas como uso eficiente da água, reaproveitamento seguro, eficiência energética, gestão de resíduos, compras sustentáveis e incorporação de tecnologias menos impactantes. Seu objetivo é servir como guia operacional, apoiando gestores e equipes na tomada de decisões baseadas em evidências.

### Implementação de Práticas Sustentáveis Nas Iniciativas Institucionais da SBN

A SBN, enquanto entidade representativa nacional, deve liderar pelo exemplo. Incorporar critérios de sustentabilidade na sua sede, em congressos, eventos científicos, reuniões e publicações reforça a coerência institucional e amplia o alcance das mensagens ambientais.

A aplicação prática inclui a redução de materiais plásticos e de materiais impressos, priorização de recursos digitais, escolha de fornecedores com práticas responsáveis e, sempre que possível, estratégias de mitigação e compensação de emissões. Essas ações alinham a atuação institucional da SBN às recomendações que propõe aos serviços assistenciais.

### Documento de Contingência Climática

Eventos extremos associados às mudanças climáticas têm impacto direto sobre a continuidade do cuidado renal. Estudos demonstram aumento da morbimortalidade em pacientes em diálise durante desastres naturais, evidenciando a necessidade de planejamento específico^5^,^6^.

Esta recomendação propõe a elaboração de um Documento Nacional de Contingência Climática em Nefrologia, com protocolos para diferentes cenários de crise. O documento deverá orientar serviços, profissionais e pacientes sobre preparação, resposta e recuperação, integrando-se a sistemas locais de saúde.

### Incentivo à Prevenção da DRC e ao Transplante Renal

Nenhuma estratégia de sustentabilidade em nefrologia é completa sem o devido investimento em prevenção da DRC e a ampliação do acesso ao transplante renal. Evidências demonstram que a prevenção, o diagnóstico precoce e o tratamento adequado da DRC reduzem a progressão para estágios avançados, diminuem a necessidade de diálise e, consequentemente, o impacto ambiental do cuidado renal^11^.

O transplante renal, além de oferecer melhor qualidade de vida e sobrevida, apresenta menor pegada ambiental quando comparado à diálise. Assim, esta recomendação reforça o papel da SBN na articulação de programas de prevenção, rastreio, diagnóstico precoce e manejo da DRC, capacitação da atenção primária, campanhas educativas e fortalecimento das políticas de doação e transplante.

## Considerações Finais

As dez recomendações aqui descritas constituem um arcabouço estratégico integrado para a construção de uma nefrologia ambientalmente sustentável no Brasil. Sua implementação deverá ser progressiva e escalonada, ao longo de um período de quatro anos (2027–2030), baseada em evidências e adaptada às diferentes realidades regionais. Os indicadores específicos de cada eixo serão discutidos, definidos e validados por um comitê de especialistas, garantindo transparência, rigor técnico e aplicabilidade.

Ao adotar essas recomendações, a nefrologia brasileira avança no alinhamento entre excelência assistencial, responsabilidade ambiental e compro­misso social, posicionando-se de forma proativa diante dos desafios impostos pelas mudanças climáticas e pela crescente carga da doença renal crônica.

## Data Availability

Os dados gerados e/ou analisados durante este estudo podem ser obtidos junto ao autor correspondente mediante solicitação razoável.
